# If We Build It, Will They Use It? Aligning Resource Recovery Design with Place-Based Social Systems

**DOI:** 10.1021/acsenvironau.5c00266

**Published:** 2026-03-02

**Authors:** Jayce D. Sudweeks, Helen Rosko, Matthew R. Landsman, Francisco Cubas, Lewis S. Rowles

**Affiliations:** † Institute for Vibrant and Engaged Communities, 7604 Georgia Southern University, Statesboro, Georgia 30458, United States; ‡ Department of Public and Non-profit Studies, 7604 Georgia Southern University, Statesboro, Georgia 30458, United States; § School of Earth, Environment and Sustainability, 7604 Georgia Southern University, Statesboro, Georgia 30458, United States; ∥ School of Sustainable Engineering and the Built Environment, Arizona State University, Tempe, Arizona 85287, United States; ⊥ Department of Civil Engineering and Construction, 7604 Georgia Southern University, Statesboro, Georgia 30458, United States

**Keywords:** resource recovery, adaptive challenges, place-based design, watershed governance, Community Capitals Framework, socio-technical systems, boundary spanning

## Abstract

Recovering water, nutrients, energy, and critical materials from waste streams is essential for building resilient and circular water systems. Yet many recovery technologies fail to achieve adoption or persistence beyond pilot scales because they overlook the social and institutional contexts that shape implementation. This Perspective reframes resource recovery as an adaptive challenge, i.e., one that depends as much on governance capacity, trust, and collective learning as on technical optimization. We propose a watershed- and place-based framework that situates recovery systems within the ecological and institutional boundaries where communities, infrastructure, and governance intersect. Within this discussion, the Community Capitals Framework offers a practical tool to operationalize places and diagnose social readiness by mapping the human, social, financial, and political assets that enable or constrain system adoption. Finally, we highlight the role of the engineer as a boundary spanner, who bridges technical and social domains to coproduce solutions that are legitimate, resilient, and locally grounded. This Perspective advances existing socio-technical approaches by integrating watershed-scale governance, place-based design, and the Community Capitals Framework into a single, operational lens that helps engineers diagnose implementation barriers and intentionally design resource recovery systems for social legitimacy, resilience, and long-term adoption.

## Graphical abstract

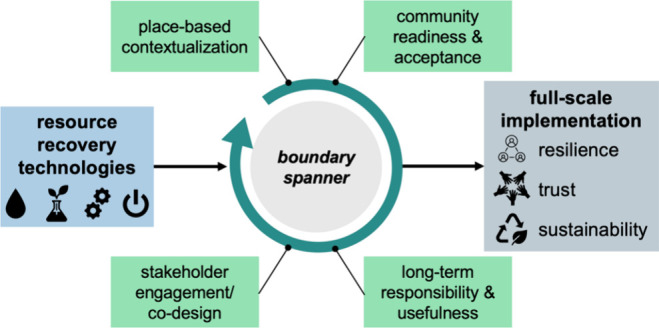

## 1 Introduction

Recovering water, nutrients, energy, and critical materials from waste streams is increasingly recognized as an important strategy for advancing resilient and circular water systems, and it contributes to multiple United Nations Sustainable Development Goals related to water security, resource efficiency, and sustainable infrastructure.^1–3^ Climate disruption, water scarcity, fertilizer volatility, and growing demand for critical materials have transformed resource recovery from an aspirational sustainability goal into a practical necessity.^4,5^ Water reuse and recycling relieve pressure on overexploited freshwater sources and buffer communities against drought, while nutrient and energy recovery reduce reliance on synthetic fertilizers and fossil fuels.^6^ At the same time, advanced electrochemical and biological processes are redefining treatment facilities as “wastewater refineries” that generate fuels and chemicals from effluents.^2^ Critical materials recovery (e.g., lithium, phosphorus, rare earth elements, and transition metals) represents an emerging frontier that introduces additional governance, market, and legitimacy challenges analogous to those observed for water, nutrients, and energy. This new frontier provides an opportunity to expedite the implementation of improved engineering design approaches that incorporate crucial socio-economic factors that promote resource recovery efforts. Together, these innovations promise to mitigate pollution, enhance resilience across the food–energy–water nexus, and close material loops critical for sustainability.^1,7–13^ Despite this technical progress, many systems that perform well in laboratories fail to achieve sustained adoption or scale once implemented.^14,15^ The reasons are rarely technical, i.e., success depends as much on public trust, political legitimacy, and governance capacity (including financial resources, regulatory authority, public perception, and organizational capability) as on reactor design or recovery efficiency.^16,17^ Traditional engineering approaches typically focus on quantifiable performance metrics (e.g., effluent quality, recovery yield, or energy balance) while overlooking the behavioral, cultural, and political dimensions that determine whether innovations are accepted and maintained. Consequently, even well-engineered projects may falter when they encounter weak institutions, fractured community networks, or cultural misalignment.^15,18^

Growing scholarship now recognizes resource recovery and reuse as socio-technical systems, i.e., networks that integrate infrastructure, policy, and social relationships.^19–21^ Within such systems, engineering decisions are inseparable from social processes, and technological outcomes are shaped by institutions, norms, and human behavior.^22,23^ This realization has spurred calls for responsible innovation, i.e., design practices that are anticipatory, inclusive, and reflexive.^24^ Embedding social scientists and community stakeholders as codesigners in engineering projects is increasingly viewed as essential to ensure that technologies align with community priorities and governance structures.^25,26^ However, engineering workflows, funding mechanisms, and education models still rarely include the structures needed to sustain such interdisciplinary collaboration.^16^ Bridging this divide therefore requires conceptual and practical frameworks that help engineers systematically evaluate the social context and translate those insights into design and implementation strategies. One promising pathway lies in place-based frameworks that couple watershed governance with community capital analysis.^27–29^ Watersheds are natural and regulatory units that organize water and nutrient management. These boundaries are not solely biophysical. Watersheds simultaneously define natural boundaries (hydrology and ecosystems), regulatory boundaries (permits, nutrient caps, and water rights), cultural and values-based boundaries (norms around water use and waste), and historical or power-based boundaries that reflect past land use, governance arrangements, and inequities. In the U.S., discharge permits, nutrient caps, and water rights are commonly defined at the watershed level.^4^ Yet legal boundaries alone are often insufficient to ensure effective or equitable implementation, particularly when policies are designed without accounting for local variation in risk, capacity, and values. Even well-crafted policy frameworks can fall short if they are not paired with mechanisms that support coordination, trust-building, and adaptation across diverse watershed actors. Within the same watershed, urban utilities, agricultural users, and industries often face distinct risks, incentives, and reuse opportunities. A watershed-scale recovery system succeeds only when these actors coordinate across political and cultural lines, i.e., when technical integration is matched by social and political integration.^30^ For example, a watershed-scale recovery system may include coordinated wastewater treatment, nutrient recovery, and reuse infrastructure distributed across multiple municipalities, agricultural users, and industrial facilities, where recovered water, nutrients, or energy are allocated based on shared watershed goals rather than isolated facility-level objectives. Thus, aligning engineered systems with watershed planning requires a deeper understanding of how local histories, power structures, and values shape technology adoption.^31^ This convergence is the essence of place-based design for engineering that explicitly accounts for both the biophysical and sociopolitical context.

The geographic concept of place offers one way to ground the broader idea that technologies are not neutral objects but social artifacts that carry and (re)­produce relationships among people, institutions, and environments.^23,32^ Considering place “socializes” technical design by situating it within the lived experiences and identities of the communities it serves.^33^ To operationalize this contextual understanding, we draw on the Community Capitals Framework (CCF).^34,35^ Originally developed in rural sociology, the CCF describes community well-being through seven interdependent forms of capital, i.e., natural, built, financial, human, social, cultural, and political. Each type of capital represents an asset base that enables and/or restricts adaptation and innovation, and investments in one can strengthen others in a “spiraling-up” dynamic.^34^ For engineers, the framework offers a systems-thinking tool to diagnose the nontechnical landscape of a project: a high-quality treatment plant (built capital) may falter without trained operators (human capital) or public confidence (social capital), while trust-building efforts can unlock funding (financial capital) and regulatory support (political capital). Conversely, emphasizing financial or built capital alone can erode cultural and social capital, undermining long-term sustainability. Applying the CCF encourages engineers to ask not merely what technology should be built but also where and under what conditions it can thrive. By mapping deficits or synergies among capitals, practitioners can anticipate obstacles and design strategies that address them. For example, a community with limited human capital may require training programs and technical assistance, while one with weak political capital may benefit from intergovernmental partnerships. This diagnostic approach transforms an abstract social context into a design parameter that can be analyzed, strengthened, and monitored throughout a project’s life cycle.^36^

Yet diagnosis alone is insufficient. Engineers must also quantify and translate contextual understanding (including social, cultural, institutional, and political factors) into a robust cost-benefit analysis to improve decision-making before and during technology design, as well as through continuous engagement and adaptive management during implementation. This role requires an expanded professional identity, i.e., the engineer must be trained and act as a boundary spanner.^37^ Boundary spanners bridge technical, governance, and community domains, facilitating dialogue and coproduction of solutions.^37,38^ They interpret complex data for nontechnical audiences, integrate stakeholder feedback into design, and mediate value conflicts inherent in adaptive challenges. By doing so, engineers strengthen social and political capitals, i.e., the relational infrastructure vital to system longevity as pipes or pumps. Rather than placing this burden solely on individual engineers, boundary spanning is most effectively achieved through intentional transdisciplinary teams that include social scientists, community representatives, and professional communicators, who can leverage their respective expertise. The engineer’s expanded identity is not about mastering all domains but about recognizing the need for and actively participating in collaborative structures where technical knowledge is integrated with social, governance, and community perspectives to address complex adaptive challenges.

Figure 1 presents the central framework of this Perspective, positioning watersheds as the integrative scale where community capitals, places, and engineered systems converge. Opportunities for resource recovery from water are embedded within the ecological and governance boundaries of a watershed, which link upstream and downstream communities, infrastructure, and institutions through shared resources and responsibilities. Within this framework, community capitals represent the assets that determine how effectively watershed-scale recovery systems can be implemented and sustained. Engineering solutions that strengthen these capitals by building trust, accountability, shared benefits, and cultural alignment can extend beyond technical performance to become enduring systems of stewardship. A watershed and place-based lens moves recovery beyond technical validity toward solutions that are socially legitimate, resilient, and supported by enduring community ownership. The contribution of this Perspective lies in intentionally synthesizing existing theories into a coherent and practice-oriented structure for environmental engineers. By coupling watershed-scale governance with the geographic concept of place to operationalize social contexts through the CCF, we provide a systematic way to diagnose social readiness and implementation risk alongside technical feasibility to sustainably incorporate resource recovery into engineering design. This integration extends prior socio-technical and responsible innovation frameworks by explicitly linking social diagnosis to engineering decision-making across design, implementation, and evaluation. In doing so, the paper reframes resource recovery beyond a technical optimization problem, to an adaptive engineering challenge requiring boundary-spanning.

**Figure 1:**
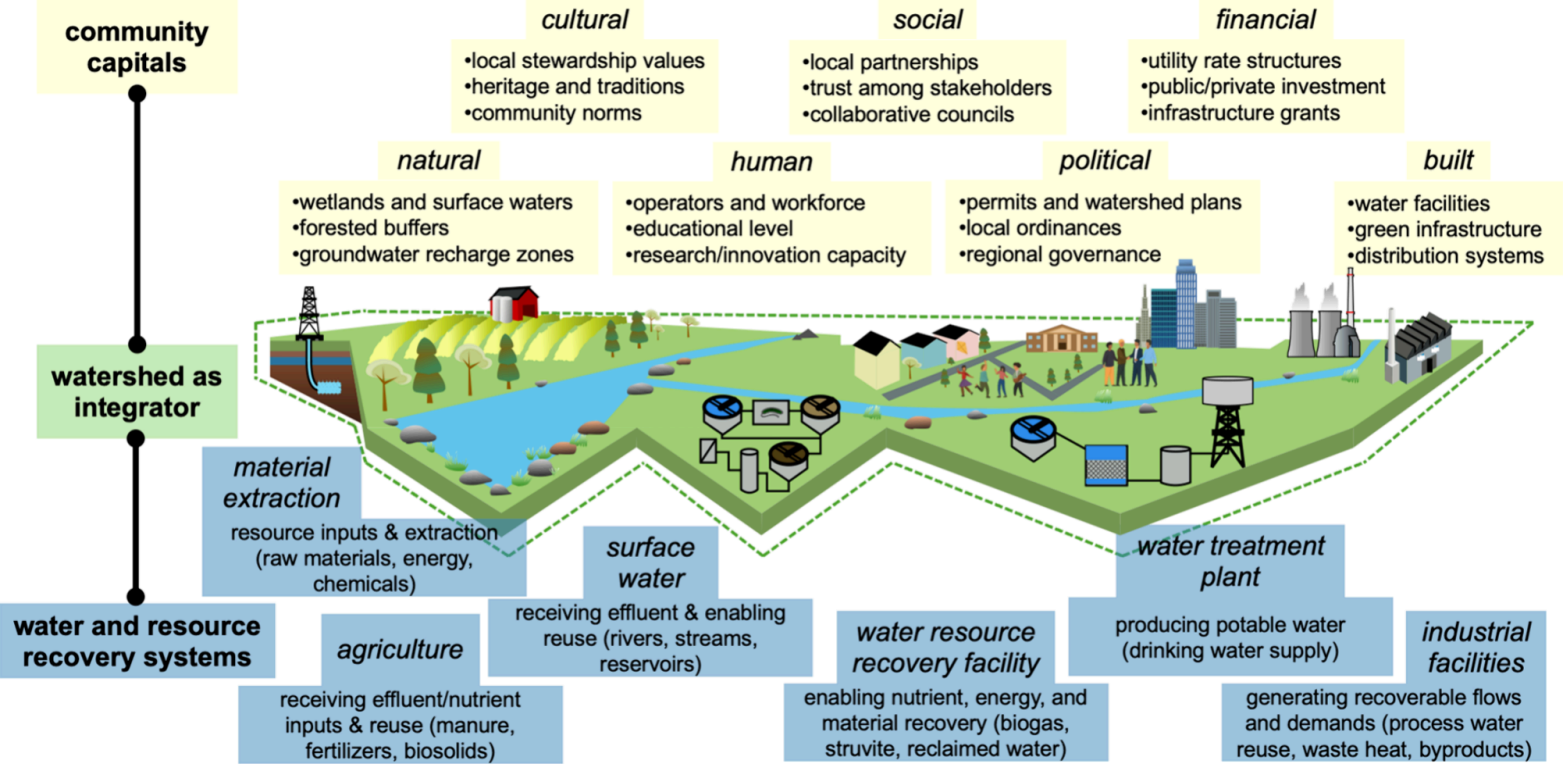
Conceptual illustration of how community capitals and place-based contexts intersect across agricultural, urban, and industrial systems to support the recovery of water, nutrients, energy, and critical materials.

## 2 Diagnosing the Failure: Technical Problems vs Adaptive Challenges

The persistent failure of technically sound resource recovery projects can often be traced to a fundamental misdiagnosis of the problem at hand. This section establishes a critical distinction between technical problems, which can be solved with existing expertise, and adaptive challenges, which require shifts in stakeholder values and behaviors.^39–41^ Correctly identifying the nature of the challenge is the essential first step for the successful design, planning, and implementation of any resource recovery initiative.

### 2.1 Technical Problems and Adaptive Challenges

When considering the types of problems that engineers are tasked with solving, it is helpful to distinguish between technical problems and adaptive challenges. A simple resource recovery analogy is instructive in understanding the differences. Designing an anaerobic digester for increased methane yield is a technical problem, a complex yet well-defined optimization task governed by established biochemical, hydraulic, and thermodynamic principles. It can involve manipulating measurable parameters such as organic loading rate, solid retention time, temperature, and reactor configuration to achieve predictable performance outcomes. It is a complicated but solvable puzzle involving known parameters and processes. In contrast, establishing a city-wide food waste collection program to supply that digester is an adaptive challenge. This challenge requires changing resident behavior, building new municipal logistics, aligning financial incentives, and fostering public trust. All of these tasks require learning and adjustment from the stakeholders themselves and not just top-down solutions from experts.

Heifetz (1994) distinguishes between technical problems and adaptive challenges.^39^ Technical problems can be solved with existing expertise, e.g., by optimizing a bioreactor. Adaptive challenges, however, require fundamental shifts in the values, beliefs, and behaviors of stakeholders, e.g., securing community trust to provide a consistent organic waste stream for that bioreactor. This framework aligns with Rittel and Webber’s (1973) concept of ‘wicked problems,’ where the problem’s definition is intertwined with the proposed solution, and stakeholders hold conflicting values about both.^42^

Adaptive challenges are complex, ambiguous, and ill-defined.^43^ They reflect broader contextual conditions, including cultural, political, institutional, and economic factors that shape how technologies are defined, implemented, and sustained.^44^ In many cases, it is not only the technology that must be adapted to place but also the implementation strategies themselves (e.g., engagement approaches, governance arrangements, financing mechanisms, and operational models) that must be tailored to local context to form an implementable intervention even in the absence of stringent regulations. In these challenges, the problem definition itself is often contentious with stakeholders holding divergent perspectives. They are marked by uncertainty and dynamism, lacking straightforward solutions and demanding learning, innovation, and a significant transformation in the mindset of those involved.^45^ A discernible gap exists between current reality and the desired state, which cannot be bridged solely by expert knowledge.

On the other hand, technical problems are often characterized by relatively well-defined system boundaries and performance objectives, allowing them to be addressed through engineering analysis and design;^45,46^ however, this does not imply that optimal or definitive technical solutions always exist, particularly in complex environmental and public health contexts. Importantly, distinguishing technical problems from adaptive challenges does not imply that technical solutions are inherently “correct” or that communities must simply adapt to them; in many cases, what is framed as a technical solution may itself be poorly defined because it was not designed with sufficient attention to the local context, values, or constraints. Technical problems are often described in the planning literature as “tame” problems^42^ because progress typically proceeds through the application and refinement of established technical approaches. In practice, this often involves engineers optimizing or adapting an existing technology through largely procedural analysis, testing, and iteration, even though uncertainty and trade-offs remain. Importantly, this does not imply that expertise is only relevant for technical problems; adaptive challenges also require distinct forms of expertise (e.g., social, institutional, and contextual knowledge) that are intertwined with technical expertise. This distinction is evident across many fields when the technical object of intervention is separated from the broader implementation challenge. In public health, for example, developing a medication that effectively targets the biological mechanisms of an infectious disease can be framed as a technical problem, whereas designing and governing an intervention to deliver that medication at scale (i.e., ensuring adherence, trust, equitable access, and supportive social conditions) constitutes an adaptive challenge. Similarly, transitioning a community to a circular economy presents a wicked problem; creating a regional system to codigest municipal biosolids with food waste involves interconnected financial, social, and political hurdles far beyond the technical design of the anaerobic digester itself. Table 1 is provided as a concise and orienting synthesis to establish shared terminology for readers across engineering and social science backgrounds and to ground the subsequent discussion of place-based implementation and community capitals.

**Table 1:** Comparison of the Defining Characteristics of Technical Problems and Adaptive Challenges in Engineering Practice^a^

**characteristic**	**technical Problem**	**adaptive challenge**
problem definition	tame problem; clear and well-understood	wicked problem; ambiguous; part of the problem itself
solution path	known and implementation by experts	unknown; requires learning, innovation, and context; and multiple perspectives
locus of work	primarily done by an authority or expert	done by the stakeholders themselves or through the transdisciplinary team-based approach
required skills	application of current expertise and resources	experimentation, collaboration, learning, and flexibility
nature of solution	straightforward, often technical fix	requires shifts in stakeholders’ values, beliefs, and behaviors

a Technical problems can be solved using established expertise and procedures, whereas adaptive challenges require stakeholder learning, behavioral change, and governance adaptation for successful implementation.

While technical problems often draw on engineering and scientific expertise, defining and addressing such problems also requires other forms of expertise, including local knowledge, operational experience, and contextual insight held by community members, practitioners, and regulators. This procedure often involves processes like adaptive management, where strategies are continuously monitored and adjusted based on feedback.^47^ Such an approach requires the active involvement of all stakeholders to cocreate solutions that are dynamic and context-aware. In addition to distinguishing between technical problems and adaptive challenges, it is important to realize that most adaptive challenges have a technical component to them. The greatest risk, therefore, often lies not in the technical problem itself but in the attempt to implement a solution without understanding the context into which it is introduced. In addition to solving the technical challenges, engineers need to understand what obstacles may impede implementation and sustainability. It is important to note that this framing is intended to distinguish types of challenges, not to privilege technical knowledge over other forms of expertise, or to suggest that social adaptation is required to accommodate predetermined technical designs. In practice, this distinction is most useful when applied iteratively, allowing engineers to revisit problem definitions, assumptions, and design choices as contextual constraints become clearer.^48,49^ Used in this way, the technical and adaptive framing supports reflective engineering practice rather than the prescriptive categorization of problems or solutions. We draw on Heifetz’s (1994) distinction between technical and adaptive challenges because it offers a clear analytic way to separate problems that can be resolved primarily through expert optimization from those that require collective learning, shifts in values, and changes in governance.^39^ Although these concepts originated in organizational leadership studies, similar distinctions have a longer lineage in environmental and sustainability scholarship, including adaptive management in environmental governance,^47^ socio-technical transition theory,^20^ and transdisciplinary sustainability science.^41^ In this Perspective, we therefore use Heifetz’s terminology as an organizing framework to highlight where implementation barriers are fundamentally social and institutional rather than purely technical, while recognizing that most real-world problems combine technical and adaptive elements. Throughout this paper, the term “expertise” is used broadly to include not only technical and scientific knowledge but also local, operational, and experiential knowledge that is essential for designing and implementing sustainable interventions.

### 2.2 Application to Resource Recovery

In environmental engineering, addressing adaptive challenges requires analyzing the entire socio-technical system, i.e., the complex web of infrastructure, environmental conditions, human behavior, financial incentives, and political structures that must be considered in unison.^19,20^ By integration of social, financial, technical, and environmental components, this approach facilitates holistic and robust solutions at the intersection of human activity and environmental sustainability. Within engineering practice, a socio-technical perspective is achieved through the principle of joint optimization.^50^ Rather than designing technical systems first and adding social considerations later, joint optimization requires developing social and technical subsystems simultaneously, ensuring that infrastructure, institutions, and human practices are codesigned to function within specific social contexts. This integrated perspective is crucial for developing solutions that are adaptable and ensure long-term sustainability.^50^ Importantly, many of these adaptive challenges manifest most clearly at the watershed scale, where regulatory authority and policies, infrastructure ownership, and material flows intersect across municipal, agricultural, and industrial actors. Watershed-scale governance structures (e.g., nutrient caps, discharge permits, best management practices, and basin-wide planning institutions) therefore shape and are shaped by technical feasibility as well as the political and social conditions under which recovery systems can be coordinated, financed, and maintained.

Unlike a purely technical problem, such as calculating the correct chemical dosage for struvite precipitation at a resource recovery facility, an adaptive challenge lacks a simple, expert-driven solution. Instead, the challenge is deeply embedded within the system’s existing structure, culture, and values. It is a recognition that environmental problems and their solutions are never purely technical; they are deeply intertwined with human behavior, societal structures, and governance. From a resource recovery perspective, the technology of resource recovery presents a technical problem; its implementation poses an adaptive challenge. Table 2 summarizes illustrative examples of resource recovery projects to distinguish between technical problems (engineering design tasks) and adaptive challenges (implementation challenges requiring social, political, and organizational alignment).

**Table 2:** Examples of Resource Recovery Projects Illustrating the Distinction between Technical Problems (Engineering Design Tasks) and Adaptive Challenges (Implementation Tasks Requiring Social and Governance Alignment)^a^

**project type**	**technical problem (the design task)**	**adaptive challenge (the implementation task)**
nutrient recovery	designing a struvite precipitation reactor to meet a target phosphorus removal efficiency	creating a viable, trusted, and sustainable market for the recovered struvite, which involves:
		• changing farmer perceptions
		• navigating fertilizer regulations
		• building new supply chains
energy recovery	optimizing an anaerobic digester to maximize methane yield from wastewater sludge	establishing a municipal codigestion program by:
		• convincing restaurants to separate food waste
		• addressing community concerns about truck traffic/odors
		• developing a use for the generated energy
water reuse	designing an advanced water treatment facility to meet state standards for nonpotable or direct potable reuse	gaining public acceptance and appreciation by:
		• overcoming the psychological “yuck factor” for potable reuse
		• establishing new water rights frameworks
		• creating a pricing structure that makes recycled water economically viable

a Each example highlights the differing competencies needed for successful project outcomes. While this framework distinguishes technical from adaptive elements for analytical clarity, it is important to recognize that problem identification itself is never neutral. It is shaped by existing power relations, cultural values, and political-economic structures. Thus, even seemingly ‘technical’ design questions reflect prior social choices about what problems matter and whose needs are prioritized.

Engineers need to recognize them as adaptive challenges. This necessitates a different approach from the engineering team, one focused on facilitation and stakeholder learning as well as attention to social, economic, and political factors that influence the design, implementation, and effectiveness of their solutions. Describing this distinction between technical solutions and adaptive challenges and the ramification is not a critique of technical expertise and current engineering practices but is a necessary expansion of the engineer’s toolkit to address the full complexity of real-world projects.

## 3 Framework for Context

Successfully addressing an adaptive challenge requires a deep understanding of the context in which a project is implemented. This section introduces the geographic concept of place as the essential framework for analyzing this context. We then present the CCF as a diagnostic tool for systematically understanding the place-based community capitals within a particular place, i.e., social, cultural, financial, natural, built, human, and political assets within that place. Through real-world examples, we demonstrate how this integrated approach can effectively diagnose the underlying reasons for the success or failure of resource recovery projects.

### 3.1 Bridging Socio-technical Relations through Place

Scholars have consistently recognized that social relations are embedded in the design and outcomes of technology; however, technical solutions are often framed and implemented as asocial or apolitical.^23,51^ The geographic concept of place provides a powerful means to address this intersection by tailoring and contextualizing technologies in more socially appropriate, sustainable, and resilient ways.^30,31^ This focus on place effectively “socializes” technical design, challenging the notion of “technological determinism” and the simple “technical fix” by demonstrating that technologies are fundamentally products of, and embedded in, broader social relations.^23,26^ In this sense, place provides a systemic lens that considers not just a location’s key material characteristics (e.g., ecologies, resources, and infrastructure) but also abstract aspects like culture, values, and meaning.^28,52^ In turn, place accounts for the biophysical, sociopolitical, and economic elements within sites of intervention that more accurately reflect the lives, identities, and practices of the people within those locations.

At the same time, place is inherently scalar (e.g., socially, spatially, and temporally) and it fundamentally shapes the success of any intervention.^28^ Encompassing localized ecology, economy, and community values, place serves as a critical component for tailoring resource recovery technologies to the appropriate scale, thus maximizing environmental and social effectiveness. In this perspective, we use the term “place” deliberately rather than “context” to emphasize the sociomaterial, spatial, historical, and institutional specificity of where interventions are implemented. While context can be abstract or generalized, place anchors of technical systems within particular watersheds, communities, infrastructures, and governance arrangements that shape both problem definition and implementation.

In water and resource recovery contexts, the watershed provides a particularly consequential scale of place because it links upstream and downstream communities through shared hydrologic, regulatory, and governance systems that shape nutrient loads, water reuse opportunities, and emerging recovery pathways. As a result, design decisions for water resource recovery facilities must account not only for technical performance but also for the livelihoods, land uses, and development trajectories that characterize a given place. For example, South Georgia, USA, is a predominantly rural region that is beginning to grapple with reuse and recovery concerns. Communities there are historically agricultural, and public infrastructure capacity may be limited; thus, one design pathway may involve a low-complexity, low-maintenance lagoon system serving a small municipality to stabilize wastewater effluent quality, reduce nutrient loads to receiving waters, and reuse treated water or biosolids for compost. In contrast, in rapidly urbanizing or industrializing watersheds, advanced treatment systems, such as the membrane bioreactor at the North Bryan County Water Reclamation Facility in Bryan County, GA, USA, may be more appropriate. This facility illustrates how treatment infrastructure can be scaled and configured to support large industrial users, as it has been designed to serve Hyundai Motorgroup Metaplant America and associated developments. In places undergoing rapid socioenvironmental transformation (e.g., South Georgia, USA, shifting from agricultural to manufacturing livelihoods), these distinctions in scale, complexity, and intended reuse pathways become especially consequential. Aligning recovery technologies with watershed-scale conditions and local socio-economic context helps ensure that systems remain technically viable and socially sustainable over time.

Using the CCF to identify local social norms and practices, place acts as the operational bridge that grounds social relations and links them to tangible resource recovery solutions. Considering place accomplishes two critical goals: it leverages local history and community experience to build the trust and acceptance necessary for long-term management; and it necessitates the inclusion of local knowledge to holistically identify needs and define beneficiaries. In this way, focusing on place mobilizes a significant shift in knowledge production, one that grounds resource recovery in local people and their environments rather than relying solely on top-down, scientific authority. Importantly, place-based evaluation may also reveal that a proposed technology is poorly aligned with local conditions and therefore requires substantive redesign or replacement, rather than simply stakeholder adaptation or governance arrangements. In this sense, attention to place supports adaptive governance by creating space for reflexive, iterative decision-making, where technologies themselves remain subject to reconsideration in light of contextual knowledge, values, and evolving societal goals.^24,26^ For environmental engineers, evaluating places can be operationalized in a team-based manner through systematic assessment of watershed-scale biophysical conditions, infrastructure configurations, governance and regulatory settings, community capacities, and historical development trajectories, which together inform feasible system designs and implementation strategies.

### 3.2 Opportunities for Place-Based Resource Recovery in Engineering

The conceptual and practical application of place through place-based interventions is well-established across disciplines including international development, public health, and urban planning.^25,53,54^ However, its role in engineering remains nascent, focusing primarily on education and learning.^27,33^ Thus, opportunities to integrate social relations rooted in a particular place into all aspects of technical solutions provide one way to improve the success and sustainability of resource recovery projects in engineering.

Engineering, in its foundational capacity, is well-suited to address the biophysical and environmental components of place. In keeping with the example provided above, water resource recovery facilities not only clean wastewater but also recover it for reuse (e.g., irrigation or indirect potable use). This recovery provides an immediate environmental benefit by reducing the reliance on finite freshwater resources, thereby aiding in the restoration of natural water balances.^55,56^ However, the full potential of this technological solution remains unrealized without the corresponding social and institutional infrastructure. Reclaimed water is valuable only if the government and community have clear pathways for accessing and using it. In many cases, advanced treatment technologies are technically sound, but absence of complementary programs, institutional arrangements, socialization programs, or delivery infrastructure (e.g., irrigation programs, industrial reuse agreements) results in underutilized reclaimed water infrastructure. In extreme circumstances, such technologies without public knowledge result in general mistrust. This type of failure is distinct from situations in which a technology itself is poorly matched to local conditions or needs; distinguishing between these mechanisms is critical for diagnosing misalignment and determining whether additional support systems or technology redesign is required.

For design, place-thinking enables intentional discussions about transdisciplinary teams that authentically integrate local knowledge, including community leaders, nonprofits, and the private sector. This ensures that the production process is informed by those most aware of local needs. Place helps to then ground the implementation of resource recovery technology, ensuring that it is technically and financially feasible, easily understood by various operators, and ready for local regulation and scale. Finally, evaluation demands a comprehensive assessment enabled by the place-context. This ensures checks like assessing community involvement to guarantee public acceptance and product adoption, and verifying that the technology’s design is flexible and scalable enough to handle future flow increases or changing regulations. We argue that making the place an integral component of resource recovery is key to ensuring its long-term success and replicability. The application of place to engineering has a broader intellectual and public impact: it helps decolonize traditional approaches by authentically valuing non-Western and nonscientific knowledge, demonstrating alternative social relationships with nature, and ultimately improving outcomes for a wider range of needs and users.^57,58^

### 3.3 Community Capitals Framework: A Tool for Issue Diagnosis

While “place” provides the essential holistic context, the CCF offers a structured methodology to systematically map the specific assets within a given place. In this Perspective, the CCF is used as a diagnostic tool that can inform multiple stages of project development, including assessment of implementation readiness, evaluation of contextual fit, and identification of likely sources of failure. Rather than serving as a predictive checklist, CCF helps engineers and planners systematically examine in the design phase how place-based capacities and constraints shape whether a resource recovery intervention can be implemented, sustained, or scaled. This framework is a systems-thinking approach used to analyze community development through an asset-based lens. Originating from rural sociology as a response to purely financial indicators of well-being, the CCF provides a holistic perspective by identifying seven interdependent types of capital: natural, cultural, human, social, political, financial, and built.^35^ Within this framework trust is not treated as social capital itself, but rather as a mechanism that generates, maintains, and mobilizes social capital by enabling cooperation, reciprocity, and durable social networks.^17–60^ As detailed in Table 3, each capital represents a category of community assets.^61,34^ Throughout this paper, terminology is aligned with the CCF, i.e., references to governance, regulation, and decision-making are treated as elements of political capital, while references to costs, markets, and funding are described as financial capital.

**Table 3:** Overview of the Seven Community Capitals Comprising the Community Capitals Framework (CCF)^a^

**capital**	**definition**	**community example**	**role in resource recovery projects**	**capital-specific actions & considerations**	**typical failure modes when capital is deficient**
natural	quality and quantity of environmental resources	clean rivers, healthy forests, fertile land, and clean air	the project’s direct impacts and benefits on the environment and natural resource base	quantifying improvements in water quality, enhancing soil health, reducing landfill burden, and lowering greenhouse gas emissions	ecological harm undermines legitimacy or regulatory approval
cultural	values, norms, beliefs, and traditions that shape a community’s identity and worldview	local festivals, historical heritage, shared language, community celebrations, ethnic traditions	the alignment of the project with the community’s values, identity, and worldview	assessing how a project aligns with local identity (e.g., a community valuing its agricultural heritage may be more receptive to biosolids application)	rejection due to perceived misalignment with community values
human	skills, knowledge, health, and abilities of residents	an educated workforce, skilled tradespeople, leadership abilities within the community	the skills, knowledge, and abilities needed from community members and project staff	training plant operators, educating the public on source separation programs, and developing local technical and managerial expertise	operational failure due to lack of expertise
social	connections, networks, trust, and reciprocity among people and organizations	volunteer groups, strong neighborhood associations, high levels of trust between residents	the networks, trust, and relationships required for project acceptance and long-term success	engaging stakeholders via public meetings, forming public-private partnerships, and building public trust in the utility and the recovered products	public opposition, misinformation, lack of participation
political	capacity to influence decision-making and shape outcomes through established governance systems and regulatory processes	participation in public hearings, access to local representatives, enforcement of zoning/building codes, voting in local elections, and lobbying for regulatory updates	the ability to navigate the policy and regulatory landscape and secure institutional support	working with regulators to permit new technologies, securing support from elected officials, and aligning with regional sustainability plans	regulatory delays, unclear authority, policy reversal
financial	financial resources available to invest in community capacity building, businesses, and development	community foundations, local banks, grants, individual wealth, and business loans	the economic aspects of the project, including funding, costs, and revenue generation	securing project financing, setting user fees, and creating stable markets for recovered products (e.g., biogas, reclaimed water, compost)	projects stall after pilots; inability to sustain operations
built	human-constructed infrastructure and facilities in a community	roads, bridges, buildings, water and sewer systems, broadband Internet access	the physical infrastructure of the project. This is the traditional domain of the engineer	design and construction of treatment facilities, pipelines, anaerobic digesters, and composting infrastructure	infrastructure underutilized, poorly maintained, or abandoned

a Each capital represents a form of community asset (natural, cultural, human, social, political, financial, and built) that collectively supports community resilience and sustainable development. The application of the CCF to resource recovery projects is shown with each capital’s role in project success and provides example actions or considerations that engineers can integrate into planning, design, and implementation processes. Definitions of the community capitals are adapted from literature.^61,34^

The framework’s central premise is that these assets are an interconnected portfolio; successful development initiatives create a positive feedback loop, or a “spiraling-up” effect, where strategic investment in one capital builds capacity in others.^34^ A community might, for instance, leverage its social capital (strong volunteer networks) and natural capital (a restored riverfront) to build financial capital through tourism and recreation.

Conversely, the interconnected nature of the capitals means that development projects often involve difficult trade-offs. An overemphasis on one form of capital can lead to the erosion of another, resulting in a negative feedback loop or a “spiraling down” effect.^34^ For example, a project designed solely to maximize financial and built capital (e.g., constructing a large waste-to-energy incinerator) may degrade natural capital through air and water pollution.^62–64^ If the decision-making process is not inclusive, then it can also erode social capital by creating community division and mistrust. Understanding these potential conflicts is crucial for engineers and planners to anticipate sources of community opposition and design more equitable and sustainable projects.

From a project management perspective, the CCF can be used diagnostically to identify capital deficits that may represent significant, often insurmountable, barriers to implementation.^65^ The absence of critical capital can render a project nonviable, regardless of its technical merits. Below are examples of how such deficits can hinder a resource recovery project:

Lack of Social Capital: A deficit in social capital is a frequent point of failure. Social capital can be subdivided into three types: bonding social capital (connections within a homogeneous group, like close neighbors), bridging social capital (connections between different groups, like environmentalists and farmers), and linking social capital (connections to those in power, like local government).^66^ A technically sound water reuse project, for example, can be halted by deep-seated mistrust between the community and the utility (low bridging social capital, which connects diverse groups). Without strong bridging networks or linking social capital (connections to those in power), unchecked misinformation can spread within homogeneous groups (high bonding social capital), making consensus building impossible.
Lack of Political Capital: A novel technology that produces a safe and effective soil amendment may never be implemented if project proponents lack the necessary political capital, i.e., the access and influence required to navigate regulatory bodies and secure approvals from state environmental and agricultural agencies.
Lack of Financial Capital: A municipal codigestion project may be technically feasible but financially nonviable if the community cannot secure low-interest loans or if a viable long-term market for the resulting biogas and digestate cannot be established to guarantee a revenue stream.
Lack of Human Capital: A small, rural community might secure a grant to build a state-of-the-art facility, but the project may fail in the long term if the community lacks the human capital needed to operate it, namely, the ability to attract, train, and retain technicians with the requisite advanced skills.

When applied in this way, deficits in specific capitals can be linked to distinct implementation failures. For example, limited financial capital may constrain long-term operations; weak political or institutional capital may delay permitting or coordination; low social capital may limit trust or participation; and insufficient human or cultural capital may hinder operation, maintenance, or appropriate use of recovered resources.

### 3.4 Application of the Community Capital Framework to Resource Recovery Projects

Engineers typically emphasize built, financial, and natural capitals by focusing on infrastructure, financial, and resource feasibility. To this end, social, human, political, and cultural capitals, which consider governance structures and community values, are equally essential to full-scale implementation, yet they often remain underemphasized in engineering design and decision-making. The CCF provides a practical typology for engineers to move beyond a more narrow focus on built, financial, and natural capital. It helps identify potential nontechnical barriers and cobenefits, allowing for a more robust and resilient project design that creates positive “spiraling-up” effects across all capitals. These dynamics extend to critical materials recovery, where technical feasibility is often outpaced by uncertainty in regulation, market formation, and public legitimacy. For example, the recovery of lithium, rare earth elements, or metals from waste streams may depend less on separation efficiency than on political capital to navigate permitting, financial capital to sustain nascent markets, and social capital to establish trust in recovered-material supply chains. Table 3 highlights the potential role of each capital in a resource recovery project, provides example actions and considerations for implementation, and summarizes typical failure modes that arise when deficits in specific forms of capital undermine project adoption or long-term performance.

#### 3.4.1 Interpreting Implementation Challenges through the Lens of Community Capitals

Table S1 in the Supporting Information (SI) presents several instances where inadequate cultural, social, and political capitals have blocked otherwise promising recovery projects. Many of these projects faced challenges not because the technologies were lacking, but because they were introduced into contexts unprepared to accommodate them.

Cultural capital deficits often emerge when recovery technologies conflict with local beliefs, practices, and identities. In Jordan, biosolids have not been adopted for agricultural reuse because they have been perceived as unsafe or impure, carrying risks of pollution and/or disease.^67^ This perception is driven, in part, by Islamic beliefs surrounding purity and waste, as well as the lack of built capital in the form of sludge stabilization technologies at many wastewater treatment plants.^68^ When cultural norms and technological capacity do not align, it is difficult to establish public confidence. It is also notable that there are ongoing scientific and regulatory discussions regarding potential environmental and public health risks associated with biosolids application to agricultural land, underscoring the importance of place-specific governance, risk assessment, and community acceptance. As a critical materials pathway, mining also illustrates how cultural and political capital shape the implementation of resource-related technologies. Similar cultural dynamics are evident in the Escobal Mine in Guatemala, which has been suspended since 2017 after the Guatemalan Constitutional court ruled out that the Indigenous Xinka communities were excluded from consultation and decision-making.^69,70^ The mining company’s failure to recognize the local community’s relationship with the land and the impacts of their project on the Xinka culture eroded trust and legitimacy.

Similarly, deficits in social capital surrounding trust, transparency, and communication have limited the success of other recovery projects. Even in the face of severe drought in 2006, community groups in Toowomba, Australia, such as “Citizens Against Drinking Sewage,” dominated national media, framing indirect potable water reuse as a health risk.^71,72^ Despite strong technical justification, interventions may struggle to gain acceptance when education and engagement efforts do not adequately address community concerns, values, and emotional responses, all of which play a legitimate role in decision-making. A similar dynamic occurred in San Diego, California (United States), in the 1990s, where the Water Repurification Project was abandoned following media backlash on the “toilet-to-tap” narrative. Subsequent reuse initiatives in San Diego gained acceptance after more than a decade of outreach, educational programs, and public facility tours, which rebuilt public confidence in local governance and water quality.^73,74^ Together, these cases highlight that social capital must be developed through long-term, participatory engagement that allows communities to “co-own” the purpose and benefits of technological change.

Deficits in political capital, including regulatory uncertainty and a lack of institutional coordination and/or accountability, further constrain the success of recovery projects. In Mpumalanga, South Africa, a mine water treatment program proposed in the early 2010s collapsed under unclear liabilities and insufficient funding for long-term maintenance.^75–77^ Disputes over the responsibility for maintenance and operation between private and public stakeholders led to the deterioration of treatment infrastructure, allowing acid mine drainage to contaminate surrounding waterways. The absence of established accountability and oversight mechanisms has led to shortfalls in political and financial capitals. Emerging contaminants and new regulatory constraints are further complicating these dynamics, such as in Maine (United States), where uncertainties over contamination by polyfluorinated compounds in biosolids led to a statewide ban in 2022 on land application of wastewater-derived fertilizers.^78^ This decision, driven by public concern and political influence, has halted nutrient recovery efforts, disrupted local markets for biosolids reuse, and reduced investor confidence and public trust in existing regulatory/liability frameworks.^79^ More broadly, the salience of particular community capitals varies by place and project, and not all capitals need to be equally engaged in every case. The CCF is therefore intended to prioritize which capitals are most critical for a given intervention in a specific context rather than function as a uniform checklist.

Across these examples, perceived risks often become salient when deficits exist in key forms of community capital. In particular, weak social capital (e.g., limited trust or past negative experiences), insufficient political or institutional capital (e.g., exclusion from decision-making), and gaps in cultural capital (e.g., misalignment with local values) can amplify concerns and undermine the perceived legitimacy of otherwise technically sound interventions. Together, these examples indicate that resource recovery developments are often adaptive challenges in which deficits in social, political, and cultural capital shape trust, legitimacy, and long-term accountability, requiring corresponding social and governance adaptation across all parties involved.

#### 3.4.2 Successful Implementation through the Lens of Community Capitals

Table S2 in the Supporting Information presents several examples in which recovery projects have achieved long-term acceptance by successfully embedding community capital in engineering innovation. These projects demonstrate how successful implementation depends on adaptive governance and boundary spanning to link technical feasibility with trust, legitimacy, and shared benefits. The following examples illustrate how successful implementation avoided the failure modes previously described by proactively building trust, aligning technologies with local values and institutions, and strengthening key forms of community capital prior to and during implementation.

National-scale initiatives for water reuse illustrate how strong social, political, and cultural capitals can normalize recovery and reuse as part of a nation’s identity. In Singapore, a densely populated island with limited natural capital for water catchment and storage, the NEWater program integrates advanced treatment with public education and branding (i.e., social and cultural capitals) to frame water reuse as a national symbol of innovation and self-reliance.^80,81^ Transparent water quality monitoring, high water quality standards, and extensive outreach have made advanced treatment technologies (e.g., membranes, advanced oxidation) visible and accessible to the general public while strengthening trust and creating jobs that promote human and built capitals.^82^ Similarly, Israel’s nationwide wastewater reuse program supplies nearly 90% of irrigation water to one of the world’s most water-scarce regions.^83^ In Israel, success was achieved through clear regulatory frameworks, equitable water allocations among farmers, and the integration of advanced treatment technologies within a transparent political structure,^84–86^ thereby fostering stability, trust, and national pride in the country’s agricultural and cultural identity. In other words, Israel has strengthened social, political, cultural, and built capitals to promote water reuse. In these two cases, widespread acceptance and legitimacy emerged from long-term, transparent efforts that, once implemented, generated additional financial and built capital (e.g., infrastructure and jobs), reinforcing social legitimacy and national urgency and pride in these engineering advancements. In contrast to the failures previously described, these national cases avoided implementation breakdown by addressing social and political capital early, ensuring transparency, participation, and institutional alignment before large-scale deployment.

City-wide efforts demonstrate how local, socially embedded capitals enable recovery technologies to achieve long-term success. In Orange County, California (United States), the Groundwater Replenishment System (GWRS) has used trust and transparency (human capitals) to transform public perception. The project has invested in social and political capitals, including proactive public education (e.g., a virtual tour available online),^87^ to keep residents informed and engaged. By emphasizing both public health and environmental benefits from the inception of the GWRS, Orange County framed potable reuse as a community solution rather than a technological risk, and consistent messaging and positive media coverage built social trust and institutional credibility.^88–90^ Comparing the success of GWRS to the failed Water Repurification Project in San Diego in the 1990s highlights the importance of community capitals in water reuse adoption. A similar outcome has been achieved by the Goreangab Direct Portable Reuse plant in Windhoek, Namibia, which has been in operation since the late 1960s and has become an indispensable resource for the city’s growth in one of the world’s most water-scarce regions.^91^ Continuous performance monitoring (built capital), alongside the locally accepted need for water reuse (i.e., water as natural capital), has reinforced political and social capitals and embedded it in their culture/society. Beyond water-scarce cities, regions that develop significant waste have also benefitted from recovery systems. In Madison, Wisconsin (United States), nutrient recovery from wastewater converts wastewater phosphorus and ammonia into a marketable fertilizer (Crystal Green), through a partnership between public utilities and private industry.^92,93^ Similar efforts are present in Austin, Texas (United States), where Dillo Dirt is produced from treated wastewater biosolids.^94^ These systems combine financial and built capitals with social and natural capitals to promote a circular economy model approved by local farmers and community members. Unlike cases where technically viable systems failed due to weak institutional support or lack of trust, these examples succeeded by coupling technical performance with sustained investment in social, financial, and built capital.

Private enterprises have also successfully implemented recovery systems in communities through similar community capital-oriented frameworks. In Eastern Finland, the closure and restoration of the Kylylahit Mine illustrates efforts to address community concerns through transparent oversight and engagement, though local debates about mining’s legacy continue. This project created political and cultural capitals in a region with a long mining history through clear and successful regulatory compliance, and in turn, is creating natural and social capitals by restoring the environment and engaging with the local community.^95–97^ Similarly, ongoing mining operations at the Cerro Verde mine near Arequipa, Peru, have fostered financial capital by providing jobs to the local community without negative social consequences.^98^ The mining company partnered with local municipal authorities to construct advanced wastewater treatment facilities so that they could reuse water in copper processing,^99^ providing political and natural capitals surrounding shared responsibilities and reducing conflict over water allocation. By providing cobenefits to the local community, this project built social and natural capitals, demonstrating the role of private–public cooperation in infrastructure investments to promote social legitimacy. Together, these initiatives illustrate that recovery and reuse can extend beyond regulatory compliance to become a means of community building and environmental stewardship.

## 4 Potential Path Forward: The Engineer as a Boundary Spanner in Transdisciplinary Teams

Having established the nature of adaptive challenges and a framework for diagnosing them, we now present a solution: an expanded role for the environmental engineer as a boundary spanner. We define this role, arguing that by developing competencies to bridge technical and social domains, engineers can navigate complexity and dramatically increase the success rate of resource recovery projects. These dynamics align with broader work on socio-technical transitions and legitimacy, which emphasizes how new technologies gain acceptance through alignment with user expectations, institutional structures, and cultural norms.^100–104^

### 4.1 Engineer as a Boundary Spanner

Environmental engineers originated from public health efforts in the 19th century, where early sanitary engineers designed water and wastewater systems to prevent disease and protect community well-being.^105,106^ From its beginnings, the field has served the public good and worked across science, policy, and society for collective benefit. That legacy positions environmental engineers as natural boundary spanners that bridge technical design with public trust, governance, and community needs. Often, environmental engineers engage in “work at the boundary,” which refers to the practice of operating at the edges of one’s team, department, organization, or field of expertise to connect with others.^107^ Boundary spanning is defined as the ability to bridge diverse stakeholder groups and knowledge domains, enabling collaborative problem-solving.^38^ The term “boundary spanners” was derived from organizational and management science and describes individuals who operate at the periphery of an organization or group, managing interactions with the external environment.^107^ A boundary spanner acts as a bridge or conduit for information and resources between different groups. They are not confined to their own silos but actively seek out and build relationships across various divides. The primary functions of a boundary spanner include the following:^108^

Information Brokerage: They gather valuable information and knowledge from external environments or other internal teams and translate it for their own group.
Coordination and Collaboration: They facilitate communication and joint efforts between different departments or even with external partners to achieve common goals.
Innovation and Idea Generation: By connecting diverse perspectives and knowledge, they identify new opportunities and spark creative solutions to problems.
Resource Acquisition: They identify and bring in valuable resources, such as new talent, funding, or strategic partnerships.

In transdisciplinary teams, engineers serve as technical liaisons between utility operators and technical stakeholders (e.g., farmers with operational knowledge), while social scientists engage with community members and the broader public and community representatives inform understanding of local values and priorities. Together, these boundary spanners bridge disparate groups (e.g., government agencies, private industry, nonprofits, and community members) with different knowledge, values, and interests, facilitating collaboration and coproduction of knowledge to address multifaceted challenges that no single group can solve alone.

### 4.2 Application to Resource Recovery

Applying the concept of boundary spanning to resource recovery reframes projects as socio-technical endeavors that require more than purely technical solutions. Within this framework, environmental engineers act as critical team members within transdisciplinary collaborations, relaying technical information about project design to social scientists and community members who possess knowledge of social and institutional contexts. This role entails a range of activities, including facilitating multistakeholder engagement, coordinating institutional actions, adapting technology to local contexts, and building community capacity to ensure system sustainability.^38,109,110^ In the context of critical materials recovery, boundary-spanning engineers may play a particularly important role in connecting utilities, regulators, and downstream manufacturers to coproduce markets and governance arrangements for recovered materials.

Table 4 summarizes example methods through which engineers can operationalize community capitals as part of a boundary-spanning practice. Participatory methods such as focus groups, stakeholder interviews, and codesign workshops are particularly effective for eliciting social, cultural, and political capitals by surfacing local knowledge, trust dynamics, and governance constraints prior to implementation.^109,111^ System mapping and causal loop diagrams support diagnosis of cross-capital interactions by visualizing feedbacks among infrastructure, institutions, and community behavior, while system dynamics modeling enables engineers to explore how policy, financing, and trust influence long-term system performance and adoption.^112^ Agent-based modeling further allows representation of heterogeneous stakeholder behavior and learning, providing insight into adoption pathways, collective action, and legitimacy formation within socio-technical systems.^113^ Together, these methods translate place-based social context into analyzable inputs that can inform engineering design choices, implementation strategies, and adaptive management over project lifecycles.

**Table 4:** Example Methods for Operationalizing Community Capitals in Resource Recovery Projects^a^

**community capital**	**general methods for operationalizing capitals**	**engineering application**
social	focus groups; participatory workshops/town halls; observations	identify trust barriers, adoption risks
cultural	codesign workshops; interviews; oral histories; observations	align technology with local values
political	stakeholder & institutional mapping; interviews; observations	diagnose governance and regulatory pathways
human	capacity assessments; training need analysis	match system complexity to operator capacity
financial	scenario analysis; system dynamics	evaluate long-term market and funding viability
cross-cutting	agent-based modeling	explore behavioral dynamics and system adoption

a Methods align with transdisciplinary research practices described in literature.^41,109–113^

Boundary-spanning in practice often takes the form of participatory and transdisciplinary engagement, approaches that are widely recognized as central to successful implementation.^37^ These processes enable the integration of multiple forms of knowledge (including system, target, and transformation knowledge) required to navigate the wicked and interdependent challenges characteristic of resource recovery.^41,115^ In this role, environmental engineers function not only as technical designers but also as integrators, who help reconcile adaptive challenges that ultimately shape long-term project viability. Even technically robust and economically viable solutions depend on social acceptance and political support to persist over time.^40,116,116,117^ Table 5 summarizes the representative contexts in which engineers perform these boundary-spanning functions across different domains.

**Table 5:** Illustrative Examples of Environmental Engineers Acting as Boundary Spanners across Technical, Social, and Governance Domains^a^

**example boundary**	**boundary-spanning role**	**key collaborators**	**relevant capital(s)**	**application example**
appropriate technology & local waste streams	applying sound engineering principles to design and build recovery systems that are scaled for community use and tailored to local waste	process engineers, academic researchers, local mechanics	built; human; cultural	building a community-scale anaerobic digester to turn food waste from local restaurants into biogas for a community kitchen, requiring engineering design for the reactor and gas collection systems
project viability & local economy	creating a sustainable financial model where the value of the recovered resource supports the project and creates local economic opportunities	electronics technicians, certified e-waste recyclers, social enterprise advisors	financial; social; political	establishing a community-run e-waste disassembly workshop that harvests valuable components for resale, requiring technical skills and a business plan to connect with the larger recycling market
community organizing & local governance	included in the problem identification as well as navigating complex regulations, codes, and public works integration	civil engineers, public health officials, municipal water department, leaders	political; social	implementing a neighborhood-scale graywater recycling system for a community garden, which requires professional engineering design, city permits, and resident agreements for a shared infrastructure; requires community involvement in problem identification and design
public education & resident participation	developing outreach and training programs to ensure the project receives a consistent and high-quality supply of materials from the community	chemical engineers, local restaurant associations, safety trainers	social; human; cultural	creating a community-scale biodiesel production facility, which requires an engineering-designed reactor and a robust participation program to collect uncontaminated waste cooking oil from local restaurants

a Each example identifies the boundary type, the engineer’s role, key collaborators, and representative applications that demonstrate how boundary spanning facilitates adaptive and sustainable resource recovery.

### 4.3 Key Competencies of an Engineer-as-Boundary-Spanner

For an environmental engineer to be an effective boundary spanner, a distinct set of competencies is required that extends beyond traditional technical expertise.^57^ A capacity for systems thinking is crucial, enabling the engineer to analyze a project through an interconnected socio-technical framework.^118^ The role also demands proficiency in communication and translation, which involves conveying complex technical concepts to nontechnical audiences and, conversely, integrating social, political, and economic concerns into the technical design.^119^ Furthermore, competence in facilitation and negotiation is essential for leading multistakeholder processes and mediating the value conflicts inherent in adaptive challenges.^120^ Ultimately, a profound sense of empathy and social awareness is essential to comprehend and appreciate the community dynamics, local history, and cultural values that are often crucial to a project’s social acceptance and long-term success.^121,108^ When engineers lack expertise in specific domains, e.g., facilitation, cultural competency, or community organizing, they must work collaboratively with professionals who possess these competencies including social scientists, community organizers, and professional communicators.

In practice, these competencies are developed through deliberate, iterative engagement with place-based tools and processes that extend beyond conventional technical training. For environmental engineers, this educational process can include the structured use of frameworks such as the CCF during early stage problem framing and intervention design, participation in transdisciplinary teams, and sustained engagement with community partners and decision-makers throughout implementation. Applied in this way, tools such as the CCF support both reflective and reflexive practice by helping engineers identify context-specific constraints, anticipate implementation challenges, and adapt technical designs and governance strategies accordingly.

## 5 Call to Action

The framework presented in Figure 2 integrates community capitals in place-based context and through an adaptive engineering cycle to describe how recovery systems achieve both technical performance and social sustainability. This integration addresses the fundamental challenge that technically viable recovery systems fail when designed as purely engineering problems rather than socio-technical interventions requiring adaptive governance and community engagement. The framework organizes three interdependent elements that collectively determine project viability: the seven community capitals (natural, cultural, human, social, political, financial, and built) representing diverse forms of value supporting system performance and legitimacy; place-based contexts existing within nested spatial scales from regional watersheds to individual sites that create implementation opportunities and constraints; and adaptive engineering cycles linking codesign, technology development, implementation, and evaluation as iterative processes. Although shown as distinct elements for clarity, codesign and engagement are intended to be integrated with technology development throughout the engineering cycle rather than treated as a separate or preliminary step. These cycles produce recoveries of water, nutrients, and critical materials while simultaneously generating feedback that strengthens social and governance foundations, with recovered resources contributing to measurable community outcomes (resilience, trust, and sustainability) that reinforce the capital from which future systems emerge. The activities shown in the interactive engineering cycle correspond directly to the boundary-spanning functions described above, including facilitating knowledge integration, coordinating across institutional and community boundaries, and iteratively adapting designs in response to place-based feedback.

**Figure 2:**
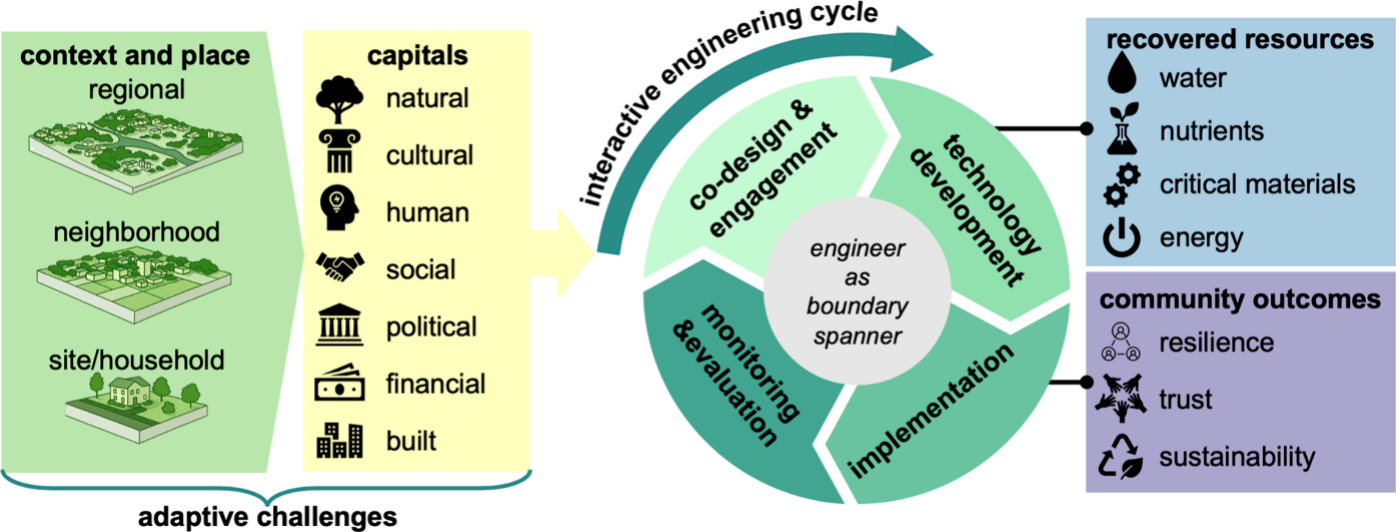
Conceptual framework linking community capitals, place-based contexts, and adaptive engineering cycles for sustainable resource recovery. Seven community capitals form the foundational inputs that guide how technologies are designed, governed, and maintained. These capitals exist within specific spatial and social contexts that shape opportunities and constraints for implementation. In this figure, “context” refers broadly to the social, institutional, and regulatory conditions influencing implementation, while “place” emphasizes the spatially and historically specific settings (e.g., site, neighborhood, watershed) in which these conditions are experienced and negotiated. The interactive engineering cycle emphasizes iterative learning and adaptation. The resulting recovery of water, nutrients, and critical materials supports community outcomes of resilience, trust, and sustainability.

Among these capitals, social capital requires particular attention as an engineered infrastructure rather than an externally determined variable. Networks of trust, participation, and shared governance enable systems to operate, adapt, and endure with the same criticality as pipes or pumps. These relational elements are essential to technology maintenance and public acceptance yet are rarely planned, funded, or evaluated with rigor comparable to that of physical components. Social capital cannot be retrofitted postconstruction but must be intentionally developed through codesign processes, transparent communication protocols, and inclusive participation structures integrated from project inception. Studies across water and sanitation contexts demonstrate that parallel social and technical design produces more durable and equitable outcomes, enabling engineers and policymakers to account for the community relationships that determine whether technologies are sustained across operational time scales.

This emphasis on social infrastructure aligns with the potential of resource recovery to address interconnected environmental and supply vulnerabilities. The recovery of water, nutrients, energy, and critical materials reduces dependence on imported materials and creates buffers against drought, energy disruptions, and material scarcity, improving resilience to climate and economic stresses when aligned with local governance capacities. However, this alignment requires parallel investment in the social and institutional systems governing technology adoption, as technical design alone cannot ensure operational longevity or public trust. Effective recovery depends on partnerships that enable communities to manage, adapt, and evolve systems in response to local needs and changing conditions.

Realizing this integration requires structural realignment of incentives within research, funding, and education systems. Funding agencies can accelerate progress by supporting projects that embed social science within engineering from conception rather than treating social analysis as supplementary validation. Success metrics must extend beyond conventional technical indicators such as treatment volumes or recovery rates to incorporate measures of social equity, political/governance capacity development, and community benefit distribution, thus better capturing project performance across technical and adaptive dimensions. Additionally, interdisciplinary training programs should prepare practitioners (i.e., both engineers and social scientists) to work collaboratively across disciplinary boundaries, engaging substantively with governance structures, behavioral systems, and cultural contexts of technology implementation. This pedagogical shift aligns innovation with responsible and adaptive design principles, where performance encompasses both technical outputs and social value generation. Sustained success of these approaches ultimately depends on aligning recovery technologies with watershed-scale institutions that coordinate actors, manage cumulative impacts, and distribute benefits and responsibilities across jurisdictions. Positive results will vary depending on the stage of implementation. For certain resources such as nutrients and energy, existing technologies can be readapted to include socio-economic aspects to improve recovery efficiency, while other areas such critical materials provide a new opportunity to implement sustainable recovery efforts bypassing challenges that were faced while recovering other constituents.

This Perspective intentionally focuses on the CCF as a practical lens for diagnosing social readiness and implementation challenges in resource recovery projects; however, it is not the only framework capable of supporting socio-technical analysis. Other approaches, such as High Reliability Organization (HRO) theory, resilience engineering, and transition management frameworks, offer complementary insights, particularly for understanding operational reliability, safety cultures, and institutional learning in complex infrastructure systems. The CCF is best viewed as a boundary-spanning diagnostic tool rather than a comprehensive theory of socio-technical change, and its value may be strengthened when used alongside frameworks that emphasize system reliability, organizational behavior, or technological transitions. Future work can explore how these frameworks can be integrated to better support adaptive governance and long-term system performance.

The framework developed in this Perspective thus provides both diagnostic and operational structure for socio-technical integration to position engineers as boundary spanners who facilitate collaboration across disciplinary and institutional boundaries, translate between technical and social domains, and coordinate stakeholder engagement throughout project lifecycles. This expanded role reflects the reality that recovery technologies succeed or fail based on alignment with community capitals and place-based contexts. Investing in social foundations at every engineering cycle stage (from initial codesign through long-term operation) determines whether resource recovery becomes transient experimentation or lasting sustainable infrastructure contributions. Rather than offering a new socio-technical model, this Perspective provides a translational framework that enables engineers to systematically connect social context, governance capacity, and community assets to concrete design and implementation choices in resource recovery projects. The challenge for the field is operationalizing this integrated approach through institutional change, educational reform, and project-level practice to treat social systems with analytical rigor equivalent to that of technical systems to address pressing environmental challenges while strengthening community resilience and sustainability.

## Supplementary Material



## Notes

Examples of failures in including cultural, social, and political capitals have blocked otherwise promising recovery projects; examples of long-term success in recovery projects by successfully embedding community capitals in engineering innovation (PDF)
