# Supplementary material for: If We Build It, Will They Use It? Aligning Resource Recovery Design with Place-Based Social Systems
Source: ACS Environ Au. 2026 Mar 2;6(3):358–74. doi: 10.1021/acsenvironau.5c00266 (PMC13195462; doi:10.1021/acsenvironau.5c00266)
Supplement: Supplementary file 1 [file vg5c00266_si_001.pdf]

**Supplementary Information for:**

# If We Build It, Will They Use It? Aligning Resource Recovery Design with Place-Based Social Systems

Jayce D. Sudweeks[1,2]; Helen Rosko[1,3]; Matthew R. Landsman[4]; Francisco Cubas[5]; Lewis S. Rowles[1,5*]

[1]Institute for Vibrant and Engaged Communities, Georgia Southern University, Statesboro, Georgia 30458, United States

[2]Department of Public and Non-profit Studies, Georgia Southern University, Statesboro, Georgia 30458, United States

[3]School of Earth, Environment and Sustainability, Georgia Southern University, Statesboro 30458, Georgia, United States

[4]School of Sustainable Engineering and the Built Environment, Arizona State University, Tempe, Arizona 85287, United States

[5]Department of Civil Engineering and Construction, Georgia Southern University, Statesboro, Georgia 30458, United States

Submitted to

*ACS Environmental Au*

*Corresponding authors: Lewis S. Rowles, email: lrowles@georgiasouthern.edu, phone: (912) 478-0772

**Table of Contents.**

**Table S1.** Examples of failures in including cultural, social, and political capitals have blocked otherwise promising recovery projects.

**Table S2.** Examples of long-term success in recovery projects by successfully embedding community capitals in engineering innovation.

**Table S1.** Examples of inadequate cultural, social, and political capitals have blocked otherwise promising recovery projects.

| case study | location | project description | key capital themes |
|---|---|---|---|
| Biosolids Land Application | Jordan | Biosolids application in agriculture has not been adopted due to perceived impurities (e.g., disease transmission) and religious concerns regarding waste products | Cultural: Religious and social norms view biosolids as impure<br>Social: Limited public education and understanding of biosolids treatment |
| Escobal Mine Suspension | Santa Rose, Guatemala | Silver mining project suspended following strong Indigenous Xinka opposition and lack of prior consultation | Cultural: Failure to recognize Indigenous rights and cultural identity<br>Political: Exclusion of affected communities from consultation and decision-making |
| Water Futures Toowoomba Referendum | Queensland, Australia | Proposed indirect potable reuse project rejected by voters in 2006 following public opposition and "toilet-to-tap" framing | Social: Lack of trust and poor risk communication surrounding "toilet-to-tap" branding<br>Political: Lack of legitimacy due to nontransparent decision-making |
| Water Repurification Project | San Diego, California, USA | 1990s potable reuse initiative abandoned amid media-driven public backlash; later revived under new governance | Social: Insufficient outreach allowed misinformation to spread to public<br>Political: Media pressure and political backdown disrupted continuity |
| Biosolids Land Application | Maine, USA | Statewide ban on wastewater-dervied fertilizers due to PFAS contamination concerns halted nutrient recovery pathways | Political: Regulatory uncertainty and unclear liability frameworks<br>Social: Public concern and risk perception outweighed technical data<br>Financial: Loss of market pathways for resource recovery |
| South Africa’s Mine Water Treatment Program | Mpumalanga, South Africa | Public-private acid mine drainage treatment initiative collapsed due to unclear liabilities, insufficient funding, and disputes over maintenance responsibilities | Political: Unclear accountability between government and private partners<br>Financial: Lack of sustained investment and funding for maintenance<br>Social: Limited community trust and transparency over environmental risks |

**Table S2.** Examples in which recovery projects have achieved long-term acceptance by successfully embedding community capitals in engineering innovation.

| case study | location | project description | key capital themes |
|---|---|---|---|
| Groundwater Replenishment System (GWRS) | Orange County, California, USA | World's largest indirect potable reuse system providing drought resilience through advanced treatment and groundwater recharge | Social/human: Long-term outreach and education built public trust<br>Political: Transparent governance/reporting strengthened legitimacy<br>Built: Advanced infrastructure demonstrated safety and reliability |
| NEWater Reclaimed Water Program | Singapore | Nationwide potable reuse program that meets the island's water demand through advanced treatment, branding, and public education | Social/human: Branding and education fostered public pride<br>Built/political: Transparent governance and performance reporting built confidence<br>Cultural: Reuse framed as a national innovation identity |
| Wastewater Reuse for Agriculture | Israel | Nationwide reuse supplying ~90% of irrigation water in a water-scarce region | Social/political: Clear regulation and equitable allocation built farmers' acceptance<br>Cultural: Branding associated reuse with national pride and self-sufficiency<br>Built/natural: Reliable infrastructure and safe water quality sustained long-term adoption |
| Goreangab Direct Potable Reuse Project | Windhoek, Namibia | Long-term potable reuse system supplies reliable drinking water in a water-scarce city | Natural: Local water scarcity drove innovation out of necessity<br>Political: Transparent regulation and monitoring built public confidence<br>Social: Reliable service long-term built community trust |
| MMSD Struvite Recovery Facility | Madison, Wisconsin, USA | Nutrient recovery facility converts wastewater phosphorus into a marketable fertilizer (Crystal Green®) | Human/social: Collaborative piloting and stakeholder engagement built local trust<br>Financial: Created local market, revenue streams, and job opportunities<br>Built/natural: Reduced maintenance costs and nutrient pollution |
| Kylylahti Mine Closure and Restoration | Eastern Finland | Tailings recovery and ecological restoration accepted due to transparent oversight and environmental compliance | Natural: Restoration improved ecological conditions<br>Political: Strong regulations ensured compliance and trust<br>Cultural: Local mining heritage fostered local understanding and acceptance |
| Cerro Verde Project | Arequipa, Peru | Mining company built advanced wastewater treatment for reuse in copper processing operations | Political: Public-private partnerships created shared accountability<br>Financial: Infrastructure investments reduced municipal and industrial conflicts<br>Social/Natural: Improved water availability for communities and environmental protection built local trust |
